# Return *Verdesmummenglaense* to the genus *Hylodesmum* (Fabaceae) based on morphological and molecular evidence

**DOI:** 10.3897/phytokeys.126.34599

**Published:** 2019-06-28

**Authors:** Zhu-Qiu Song, Gang Yao, Bo Pan, Jian-Wu Li, Xiang-Xu Huang, Yun-Hong Tan, Dong-Xian Xu

**Affiliations:** 1 Key Laboratory of Plant Resources Conservation and Sustainable Utilization, South China Botanical Garden, Chinese Academy of Sciences, Guangzhou 510650, China South China Botanical Garden, Chinese Academy of Sciences Guangzhou China; 2 South China Limestone Plants Research Centre, College of Forestry and Landscape Architecture, South China Agricultural University, Guangzhou 510642, China University of Chinese Academy of Sciences Beijing China; 3 Center for Integrative Conservation, Xishuangbanna Tropical Botanical Garden, Chinese Academy of Sciences, Menglun, Mengla 666303, China South China Limestone Plants Research Centre, College of Forestry and Landscape Architecture, South China Agricultural University Guangzhou China; 4 Guangdong Provincial Key Laboratory of Silviculture, Protection and Utilization, Guangdong Academy of Forestry, Guangzhou 510520, China Xishuangbanna Tropical Botanical Garden, Chinese Academy of Sciences Mengla China; 5 University of Chinese Academy of Sciences, Beijing 100049, China Protection and Utilization, Guangdong Academy of Forestry Guangzhou China

**Keywords:** China, Leguminosae, taxonomy, systematic position, *trnL-F*, phylogeny

## Abstract

*Verdesmummenglaense* (C. Chen & X. J. Cui) H. Ohashi & K. Ohashi is a rare species in the tribe Desmodieae (Fabaceae) from Southwest China. The morphological observation shows that the species has minute capitate stigma and ebracteolate calyces, which are entirely different from the funnel-shaped stigma and bracteolate calyces of the genus *Verdesmum* H. Ohashi & K. Ohashi, but are consistent with those of the genus *Hylodesmum* H. Ohashi & R. R. Mill. The generic placement of *V.menglaense* within *Hylodesmum* was further supported by molecular evidence. Therefore, this species should be returned to *Hylodesmum* as *H.menglaense* (C. Chen & X. J. Cui) H. Ohashi & R. R. Mill. A full description including floral characters, a colour plate and a distribution map are first provided here for this species. After excluding the solo representative in China, *Verdesmum* should be removed from the record in *Flora of China*.

## Introduction

*Verdesmum* H. Ohashi & K. Ohashi is a newly established genus in the tribe Desmodieae (Fabaceae) (Ohashi and Ohashi 2012a; Lewis et al. 2013; Azani et al. 2017), based on only one species *V.hentyi* (Verdc.) H. Ohashi & K. Ohashi which is a shrub distributed in Papua New Guinea and Malaysia and was first described in the genus *Desmodium* Desv. as *D.hentyi* Verdc. (Verdcout 1977; Ohashi 2004). This genus was considered to be most similar to *Hylodesmum* H. Ohashi & R. R. Mill, but differs in having funnel-shaped terminal stigma, bracteolate calyces, linear pods, very narrow obovate-elliptic articles and stipes longer than fruiting pedicels (Ohashi and Ohashi 2012a). Amongst these characters, the shape of the stigma was considered to be the most important trait of *Verdesmum* and to be unique amongst the whole tribe Desmodieae (Ohashi and Ohashi 2012a, 2013).

*Verdesmummenglaense* (C. Chen & X. J. Cui) H. Ohashi & K. Ohashi, the second species recognised in *Verdesmum* (Ohashi and Ohashi 2013), was originally published in the genus *Podocarpium* (Benth.) Y. C. Yang & P. H. Huang as *P.menglaense* C. Chen & X. J. Cui, based on two fruiting gatherings from Yunnan, Southwest China (Cui et al. 1987). The species had been suggested for transfer to the genus *Desmodium* Desv. by Ohashi (1995), but was not accepted by other taxonomists. Later, Ohashi and Mill (2000) found that *Podocarpium* was an illegitimate generic name and thus proposed to replace it by *Hylodesmum* H. Ohashi & R. R. Mill. Correspondingly, *P.menglaense* was proposed as *Hylodesmummenglaense* (C. Chen & X. J. Cui) H. Ohashi & R. R. Mill (Ohashi and Mill 2000; Gao 2006; Zhu et al. 2007; Huang and Ohashi 2010). The species was further transferred to the genus *Verdesmum* as *V.menglaense* (C. Chen & X. J. Cui) H. Ohashi & K. Ohashi for the similarity in the linear pods and very narrow obovate-elliptic articles (Ohashi and Ohashi 2013). This treatment was followed by subsequent research (Zhu 2015a, Zhu 2015b; Liu et al. 2015; Ohashi et al. 2018b).

**Figure 1.**
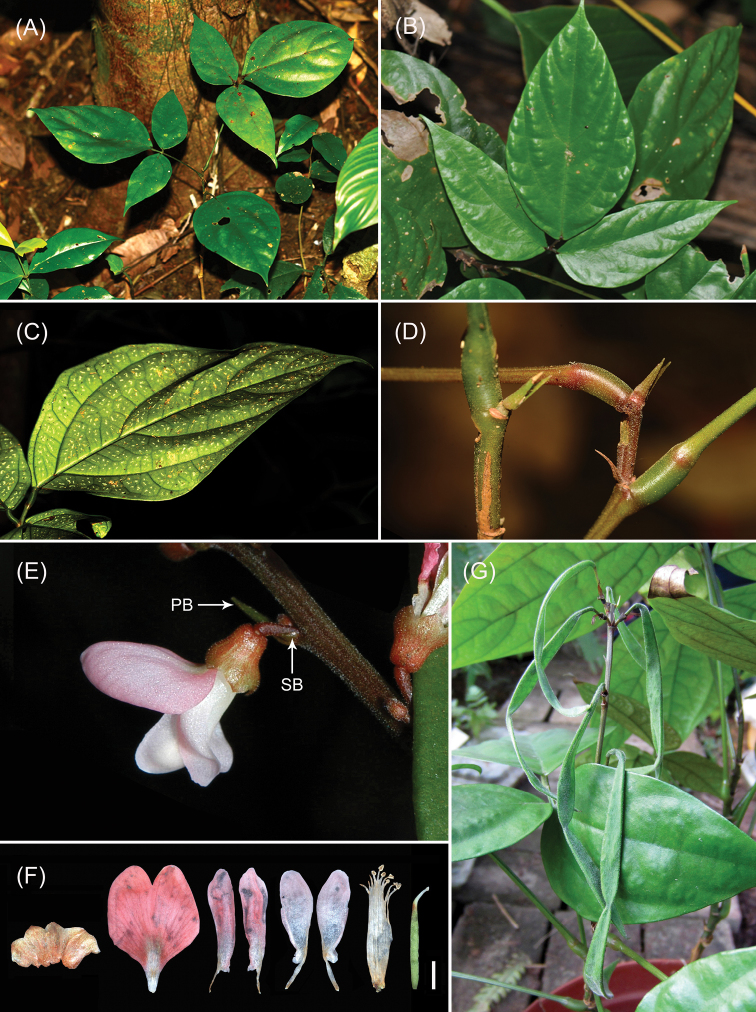
*Hylodesmummenglaense* (≡*Verdesmummenglaense*). **A** habitat **B** adaxial leaf surface **C** abaxial leaf surface, showing scattered white spots **D** stipules **E** a node of inflorescences, showing a primary bract (**PB**) and a secondary bract (**SB**), but without bracteoles at base of calyx, (**F**) a flower with the different parts separated, especially showing the terminal minute capitate stigma of the ovary, (**G**) linear pods with very narrow obovate-elliptic articles, bar = 2 mm.

**Figure 2.**
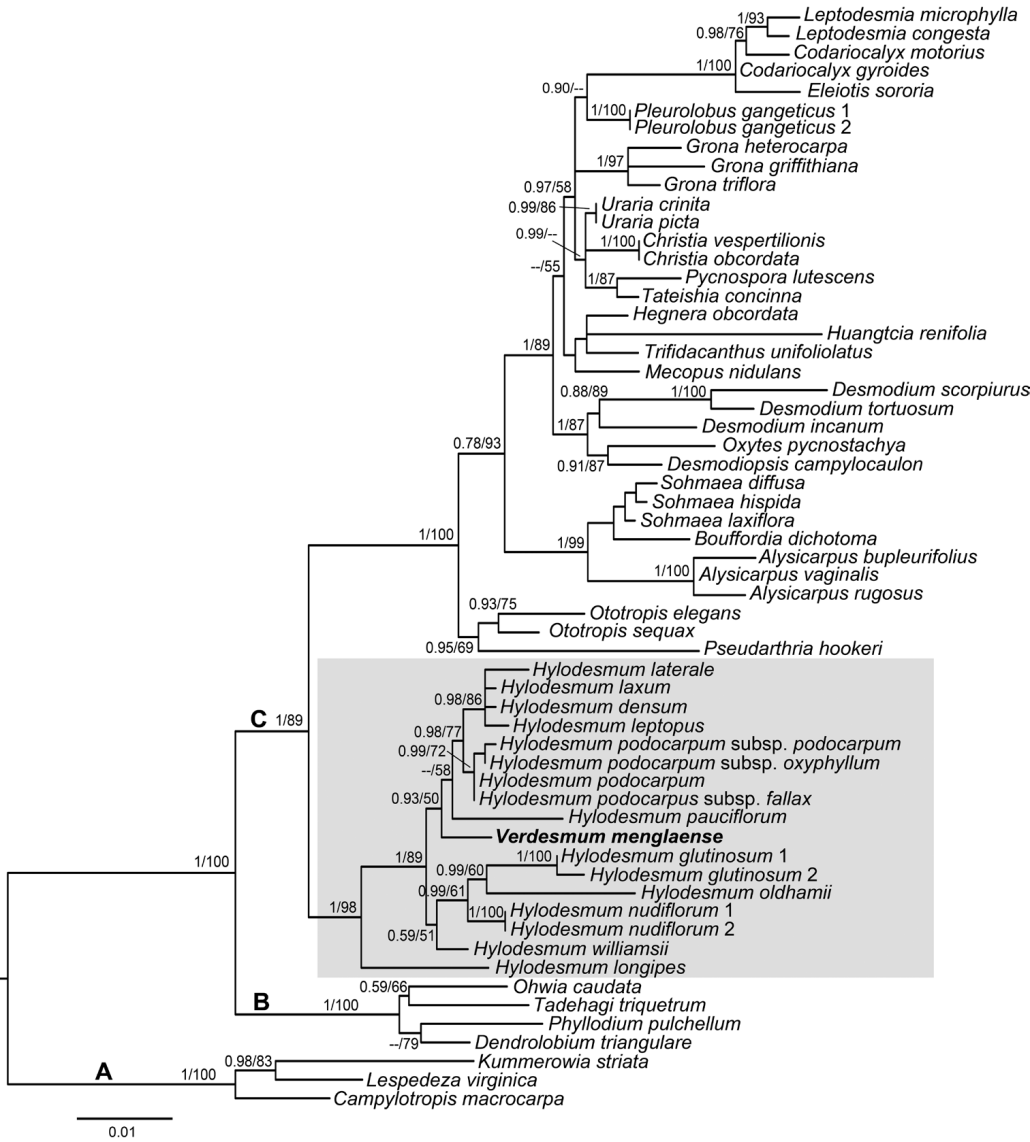
Phylogenetic relationships amongst 53 species from 30 genera of the tribe Desmodieae based on the *trnL-F* sequence data using Maximum Likelihood analysis. Numbers at the nodes are posterior probabilities and bootstrap percentages (PP, BP) from Bayesian and Maximum Likelihood analysis, respectively. A dash (--) indicates PP < 0.5 or BP < 50%. The grey cover shows the representative of *Hylodesmum* within which *Verdesmummenglaense* (indicated by bold font) was deeply embedded.

However, *Verdesmummenglaense* is a rare species endemic to Yunnan, Southwest China. After being published, it was not re-discovered in the field and its flowers have not been described in any literature (e.g. Ohashi 1995; Gao 2006; Huang and Ohashi 2010). Ohashi and Ohashi (2013) considered that it is difficult to determine the correct generic position of this species with the absence of flowers. Fortunately, in a collecting trip to Yunnan Province in 2010, we found several living individuals without flowers or fruits of this species at a streamside in the forest. Subsequently, these plants successfully produced flowers and fruits under cultivated conditions in South China Botanical Garden. Our morphological observation showed this species has terminal minute capitate stigma and ebracteolate calyces (Fig. 1). In these important floral characters, *Verdesmummenglaense* is thus distinct from *Verdesmum*, but is consistent with *Hylodesmum*. Furthermore, the placement of this species within *Hylodesmum* was also strongly supported by molecular evidence from the *trnL-F* sequences (Fig. 2). Therefore, this species should be returned to *Hylodesmum* as *H.menglaense*. Currently, *Verdesmum* just includes a single species (*V.hentyi*) and its distribution in China should be excluded.

## Material and methods

### Morphological studies

The morphological characters were examined based on the living plants and specimens kept in the HITBC, IBSC and KUN herbaria. Acronyms for the herbaria follow the Index Herbariorum (Thiers 2018). The distribution map was made by the software ArcGIS 10.2.

### Molecular analyses

In order to clarify the generic position of the species *Verdesmummenglaense* within the *Desmodium* group of the tribe Desmodieae, a phylogeny was reconstructed based on analyses of the noncoding plastid marker *trnL-F*, which was often used in phylogenetic studies of this tribe in single or combined analyses with other DNA sequences (e.g. Stefanovic et al. 2009; Nemoto et al. 2010; Xu et al. 2012; Ohashi et al. 2018b). DNA sequences were downloaded mostly from Genbank (www.ncbi.nlm.nih.gov/Genbank) and 14 taxa were newly sequenced in the present study. In total, 53 species were sampled in phylogenetic analyses, including 23 of the 28 genera in the *Desmodium* group (Ohashi et al. 2018a) and 11 of the 12 species in the genus *Hylodesmum* (if *Verdesmummenglaense* is not considered). Information about relevant samples and Genbank accessions are listed in Appendix 1. The phylogenetic trees were reconstructed using two approaches: Maximum Likelihood (ML) and Bayesian Inference (BI). Detailed information about the experiment operations (DNA extraction and PCR amplification), sequences of primer used, model selection of the sequence matrix constructed and methods in tree reconstruction can be accessed in Li et al. (2009) and Yao et al. (2016).

## Results and discussion

Results from phylogenetic analyses revealed that three groups (clade A: *Lespedeza* group, clade B: *Phylloddium* group and clade C: *Desmodium* group) were well supported in the tribe Desmodieae, just as reported in most recent research (Jabbour et al. 2018; Zhang et al. 2018; Ohashi et al. 2018a; Ohashi et al. 2018b). Although the type species of the genus *Verdesmum* was not sampled and thereby its phylogenetic position could not be resolved, the species *V.menglaense* was deeply embedded within the genus *Hylodesmum* in both of the ML (BS=98%) and BI (PP=1.00) analyses (Fig. 2). Thus, the taxonomic status of *V.menglaense* within the genus *Hylodesmum* was strongly supported by this molecular evidence, despite the absence of a good specific relationship.

Currently, *Hylodesmum* comprises 13 species (including *H.menglaense*) and 4 subspecies (Ohashi and Mill 2000; Huang and Ohashi 2010), after excluding *H.dolabriforme* (Benth.) H. Ohashi & R. R. Mill (as a member of *Monarthrocarpus*, Ohashi and Ohashi 2012b) and H.laxumsubsp.falfolium (H. Ohashi) H. Ohashi & R. R. Mill (as a synonym of H.laxumsubsp.laxum, Song et al. 2013). The genus is disjunctly distributed in eastern North America (3 species) and eastern Asia (10 species), one of which extends from Asia to Africa (Ohashi and Mill 2000; Woods 2008). China has the highest species richness in the genus and includes 10 species and 4 subspecies (Huang and Ohashi 2010; Song et al. 2013). Morphologically, *H.menglaense* is most similar to *H.leptopus* (A. Gray ex Benth.) H. Ohashi & R. R. Mill, as pointed out by Cui et al. (1987) and Ohashi (1995), because both species have calyx lobes much shorter than tube, lateral veins of leaflets not reaching margin and abxial surfaces of leaflets scattered with white spots. Especially, the white spots appear on the abxial blades only for the two species in the whole genus. However, *H.menglaense* has very narrow obovate-elliptic articles and pods with central isthmi between the articles, which are unique amongst the genus (Ohashi and Ohashi 2012a). When without fruits, we found that *H.menglaense* can be distinguished from *H.leptopus* by slightly larger and thicker terminal leaflets.

## Taxonomic treatment

### Hylodesmum menglaense

Taxon classificationPlantaeFabalesFabaceae

(C. Chen & X. J. Cui) H. Ohashi & R.R. Mill, Edinburgh J. Bot. 57(2): 180. 2000

Figure 1

Podocarpium menglaense C. Chen & X. J. Cui, Acta Bot. Yunnan. 9(3): 305. fig. 1. 1987. ≡ Desmodiummenglaense (C. Chen & X. J. Cui) H. Ohashi, J. Jap. Bot. 70(3): 142. 1995. ≡ Verdesmummenglaense (C. Chen & X. J. Cui) H. Ohashi & K. Ohashi, J. Jap. Bot. 88(3): 161. 2013, **syn. nov.**

#### Type.

China. Yunnan Province, Mengla County, Menglun Town, 21°58’N, 101°15’E, 620 m a.s.l., 6 Aug 1974, *Guo-Da Tao 009050* (holotype, HITBC!, [No. 020113]; isotype, HITBC!, [No. 020112]).

#### Description.

Perennial herbs or subshrubs, 30–100 cm high. Stem erect, simple, usually woody at base. Stipules striate, lanceolate, 3.5 mm × 1 mm in size, green to brown, uncinate-hairy. Stipels subulate, ca. 1.4 mm long. Leaves 3-foliolate, scattered along stem; petiole 8–12 cm including rachis 1–2.5 cm long, uncinate-hairy; leaflet blades thickly papery to subleathery; adaxial surfaces dark green, shiny, glabrous; abaxial surfaces pale green, scattered with white spots, very sparsely uncinate-hairy under the microscope; terminal leaflet ovate, 12–19 cm × 7–10 cm in size, entire along margin, rounded or broadly cuneate at base, acuminate or caudate at apex, 2-stipellate at base of pulvinule; lateral veins about 5 pairs, not reaching margin; lateral leaflets slightly smaller, narrowly ovate to lanceolate, base oblique, 7–12 cm × 3–5 cm in size, sessile but pulvinule distinct, 1-stipellate at base of pulvinule; pulvinule ca. 5 mm long. Inflorescences terminal or axillary, sometimes borne at leafless nodes or near the base of old stem, pseudoracemose, up to 15–50 cm long, laxly flowered, 2-flowered per node, with minute hooked hairs. Primary bracts subtending the secondary bracts, narrowly triangular, acute at the apex, 4.3 mm × 1.6 mm in size, with uncinate hairs. Secondary bracts triangular, 1 mm × 0.7 mm in size, with uncinate hairs. Bracteole absent at base of calyx. Pedicles 2.5–3 mm long, with minute uncinate hairs. Calyx 4-lobed; tube 2.5–2.6 mm long; lobes much shorter than the tube, upper lobe minutely 2-toothed at the apex, lateral and lower lobes shallowly triangular with minute hooked hairs, 1.3–1.5 mm × 0.4–0.6 mm in size; floral disc absent. Corolla pale reddish-pink, glabrous; standard blade orbiculate or suborbiculate, 7.3 mm × 6.5 mm in size, reflexed, emarginate at the apex, suddenly cuneate to the base, with ca. 1.8 mm long claw; wings narrowly elliptic, 7.3 mm × 1.8 mm in size, slightly twisted, obtuse at the apex, slightly auriculate at the base, with ca. 1.8 mm long claw; keel-petals connate, 6.6 mm × 2.3 mm in size, obtuse at the apex, auriculate at the base, with ca. 2.3 mm long claw. Stamens 10, monadelphous, filaments connate into a tube, ca. 9 mm long. Ovary linear, minute uncinate-hairy, about 7.5 mm long including style 1.5 mm long, usually 2–5-ovuled, with a very short stipe; style curved upwards, with a terminal minute capitate stigma. Pods 2–5-jointed, linear, densely minute hooked hairy, with central isthmi between articles; fruiting pedicles 5–7 mm long, fruiting stipes 9–15 mm long; articles very narrow obovate-elliptic, 3.2–5.4 cm × 3.5–6 mm in size, covered with prominent reticulate veins when mature. Seeds 1 in each locule, very narrow obovate-elliptic, 2.5–3.5 cm × 3 mm in size, without rim-arillate around the hilum.

#### Distribution.

**Figure 3.**
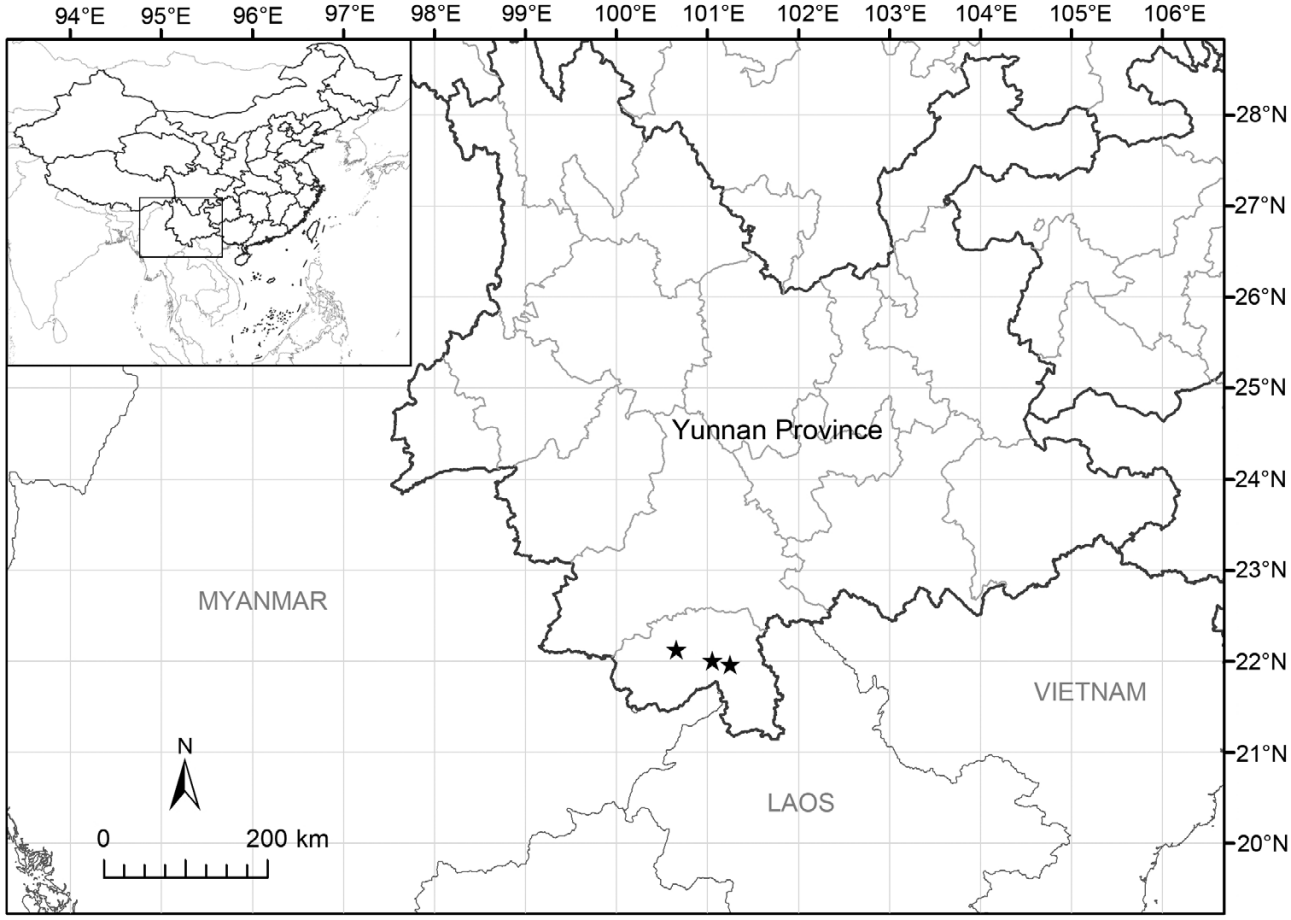
Distribution map of *Hylodesmummenglaense* (stars).

Three locations of Yunnan Province, Southwest China, were found for this species (Fig. 3). It usually occurs in moist conditions under the evergreen forests, with an elevation range from 600 m to 1000 m.

#### Phenology.

Under natural conditions in the field, the species was recorded in fruit from August to November. Under cultivated conditions in Guangzhou City, plants were observed in flower in October and in fruit from November to December.

#### Conservation status.

Before our investigation, only five type specimens of two fruiting gatherings have been found in a single locality for this species. We explored the type locality of this species and found two additional localities, but individual numbers of each of the three populations were discovered to be less than 30. Therefore this species might be considered as ‘Critically Endangered’ (CR) under the IUCN (2001) categories criteria C2a(i).

#### Notes.

*Hylodesmummenglaense* was described as having terminal and/or axillary inflorescences in some references (Cui et al. 1987; Gao 2006; Huang and Ohashi 2010). Through examining type specimens, however, Ohashi and Ohashi (2013) pointed out that inflorescences of this species arise from leafless nodes or from the base of old stem, but seem not to be terminal. Our observations show this species does produce terminal inflorescences as well.

#### Specimens examined.

CHINA. Yunnan Province: Mengla County, Menglun Town, 15 Nov 1984, 620 m a.s.l., *Xian-Ju Cui & Guo-Da Tao* 84111501 (KUN); Jinghong City, Jiluo Town, 995 m a.s.l., 11 Sept 2010, *Zhu-Qiu Song 2010091101* (IBSC); Jinghong City, Gasa Town, 24 Dec 2017, 729 m a.s.l., *Zhu-Qiu Song 2017017* (IBSC).

## Supplementary Material

XML Treatment for Hylodesmum menglaense

## Appendix 1

### Scientific names, GenBank accession numbers and voucher information for *trnL-F* used in the molecular analyses

*indicates the taxon was newly sequenced in the present study

*Alysicarpusbupleurifolius*, LC378320, T. C. Huang & M. J. Wu 14826 (TUS); *Alysicarpusrugosus*, LC378324, L. S. Man 087879 (TUS); *Alysicarpusvaginalis*, LC378321, K. Yonekura et al. 98078 (TUS); *Bouffordiadichotoma*, LC378333, L. S. Man 087882 (TUS); *Campylotropismacrocarpa*, JN402864, B. Xu & L. B. Zhang 97; *Christiaobcordata*, LC378328, K. Yonekura 12047 (TUS); *Christiavespertilionis*, LC378326, K. Ohashi 2981 (TUS); *Codariocalyxgyroides*, LC378325, K. Yoda et al. 9614111 (TUS); *Codariocalyxmotorius*, LC378327, J. M. Hu & K. H. Wang 849 (TUS); *Dendrolobiumtriangulare*, MK652468, Z. Q. Song 2010120402 (IBSC)*; *Desmodiopsiscampylocaulon*, LC378355, R. Pullen 9274 (TUS); *Desmodiumincanum*, LC378339, Yonekura 9667 (TUS); *Desmodiumscorpiurus*, LC378348, T. C. Huang & W. T. Huang 14490 (TUS); *Desmodiumtortuosum*, LC378349, H. Ohashi et al. 9580602-1 (TUS); *Eleiotissororia*, LC378354, J. Murata et al. 24817 (TUS); *Gronagriffithiana*, LC378318, K. Iwatsuki et al. 1634 (TUS); *Gronaheterophylla*, LC378338, Huan-Yu Chen 1544 (TUS); *Gronatriflora*, LC378351, K. Yonekura & K. Yasuda 11200 (TUS); *Hegneraobcordata*, LC378356, Poilane 22850 (TUS); *Huangtciarenifolia*, LC378346, N. Sasamoto 902081 (TUS); *Hylodesmumdensum*, MK652461, Z. Q. Song 90 (IBSC)*; *Hylodesmumglutinosum* 1, KM098856; *Hylodesmumglutinosum* 2, EU717294, Ellsworth 60 (IND); *Hylodesmumlaterale*, MK652460, Z. Q. Song 72 (IBSC)*; *Hylodesmumlaxum*, MK652462, Z. Q. Song 37 (IBSC)*; *Hylodesmumleptopus*, MK652463, Z. Q. Song 21 (IBSC)*; *Hylodesmumlongipes*, MK652467, Z. Q. Song 142 (IBSC)*; *Hylodesmumnudiflorum* 1, EU717296, Stefanovic SS-03-22 (TRTE); *Hylodesmumnudiflorum* 2, KM098857; *Hylodesmumoldhamii*, MK652465, Z. Q. Song 2010092801 (IBSC)*; *Hylodesmumpauciflorum*, EU717297, Stefanovic SS-03-27, (TRTE); *Hylodesmumpodocarpum*, LC378358, H. Ohashi 68914 (TUS); Hylodesmumpodocarpumsubsp.fallax, MK652459, Z. Q. Song 2010092802 (IBSC)*; Hylodesmumpodocarpumsubsp.oxyphyllum, MK652458, Z. Q. Song 2010102001 (IBSC)*; Hylodesmumpodocarpumsubsp.podocarpum, MK652457, Z. Q. Song 2010082803 (IBSC)*; *Hylodesmumwilliamsii*, MK652466, Z. Q. Song 2010082001 (IBSC)*; *Kummerowiastriata*, JN402866, N. C. Henderson 04-01; *Leptodesmiacongesta*, LC378360, E. Barnes 5 (A); *Leptodesmiamicrophylla*, LC378343, Bai-Zhong Xiao 4362 (TUS); *Lespedezavirginica*, JN402855, L. B. Zhang 4816; *Mecopusnidulans*, LC378361, Y. Tateishi et al. 1025001 (TUS); *Ohwiacaudata*, LC378362, Murata and Mori 88083 (TUS); *Ototropiselegans*, LC378363, H. Ohashi 721015 (TUS); *Ototropissequax*, MK652456, Z. Q. Song 112 (IBSC)*; *Oxytespycnostachya*, LC378345, H. S. McKee 45980 (TUS); *Phyllodiumpulchellum*, MK652469, Z. Q. Song 2010111701 (IBSC)*; *Pleurolobusgangeticus* 1, LC378336, N. Sasamoto 80201 (TUS); *Pleurolobusgangeticus* 2, LC378314, K. F. Chung 1148 (TUS); *Pseudarthriahookeri*, LC378365, K. Ohashi s.n. (TUS); *Pycnosporalutescens*, LC378364, T. Y. Liu 1202 (TUS); *Sohmaeadiffusa*, LC378334, T. C. Huang et al. 14456 (TUS); *Sohmaeahispida*, LC378357, L. S. Man 091650 (TUS); *Sohmaealaxiflora*, LC378341, N. Sasamoto 809254 (TUS); *Tadehagitriquetrum*, KF621117, X. Y. Zhu 2009052-1 (PE); *Tateishiaconcinna*, LC378317, M. Suzuki et al. 9160908 (TUS); *Trifidacanthusunifoliolatus*, LC378367, Y. Tateishi et al. 1020113 (TUS); *Urariacrinita*, LC378368, N. Sasamoto 809254 (TUS); *Urariapicta*, LC378370, M. Suzuki et al. 9191248 (TUS); *Verdesmummenglaense*, MK652464, Z. Q. Song 2010091101 (IBSC)*.
